# Establishment of appropriate glaucoma models using dexamethasone or TGFβ2 treated three-dimension (3D) cultured human trabecular meshwork (HTM) cells

**DOI:** 10.1038/s41598-021-98766-3

**Published:** 2021-09-29

**Authors:** Megumi Watanabe, Yosuke Ida, Hiroshi Ohguro, Chiaki Ota, Fumihito Hikage

**Affiliations:** Department of Ophthalmology, School of Medicine, Sapporo Medical University, Sapporo, Japan

**Keywords:** Cell biology, Eye diseases

## Abstract

To establish appropriate ex vivo models for a glaucomatous trabecular meshwork (TM), two-dimensional (2D) and three-dimensional (3D) cultures of human trabecular meshwork cells (HTM) were prepared in the presence of 250 nM dexamethasone (DEX) or 5 ng/mL TGFβ2, and characterized by the following analyses; transendothelial electrical resistance (TEER) measurements, FITC dextran permeability, scanning electron microscopy and the expression of the extracellular matrix (ECM) including collagen (COL)1, 4 and 6, and fibronectin (FN), α-smooth muscle actin (α-SMA), tissue inhibitor of metalloproteinase (TIMP)1–4, and matrix metalloproteinase (MMP)2, 9 and 14. DEX and TGFβ2 both caused a significant increase or decrease in the TEER values and FITC dextran permeability. During the 3D spheroid culture, DEX or TGFβ2 induced a mild and significant down-sizing and an increase in stiffness, respectively. TGFβ2 induced a significant up-regulation of COL1 and 4, FN, α-SMA, and MMP 2 and 14 (2D) or COL1 and 6, and TIMP2 and 3 (3D), and DEX induced a significant up-regulation of FN (3D) and TIMP4 (2D and 3D). The findings presented herein indicate that DEX or TGFβ2 resulted in mild and severe down-sized and stiff 3D HTM spheroids, respectively, thus making them viable in vitro HTM models for steroid-induced and primary open angle glaucoma.

## Introduction

The only evidence-based therapy for the treatment of glaucomatous optic neuropathy (GON) is decreasing the intraocular pressure (IOP) to suitable levels by the administration of an anti-glaucoma medication, laser treatment or surgery^1–4^. IOP levels are precisely regulated and maintained by homeostasis of the balance between the aqueous humor (AH) production and drainage through (1) the trabecular meshwork (TM) and Schlemm's canal route and (2) the uveoscleral route in which approximately 70–90% and 10–30%, of the AH is drained, respectively^5^. As a possible mechanism responsible for the increase in IOPs, resistance to elevated AH outflow by the TM caused by the excess deposition of extracellular matrix (ECM) molecules such as collagens (COLs), fibronectin (FN) and others appears to be primarily involved in the etiology of both primary open angle glaucoma (POAG) as well as steroid-induced glaucoma (SG)^6^. It has been reported that, based upon several studies using animal model as well as TM cell culture, such excess deposition of ECM could be mediated by elevated levels of transforming growth factor β2 (TGFβ2) in AH in response to treatment with glucocorticoids^7–10^.

To replicate POAG and SG pathogenesis, in vitro cell cultures using human TM (HTM) have recently been used to examine the effects of TGFβ2 as well as dexamethasone (DEX) on their efficacies on transcellular pressure and ease of outflow^8–10^. The molecular mechanisms responsible for the pathological changes in glaucomatous TM as well as the efficacy of several anti-glaucoma medications have been studied using these models^11^. However, most of these studies have used conventional 2D cell cultures, although the HTM is composed of multiple sheets^12^. Therefore, to replicate the 3D structure of the human TM, a relevant 3D cell culture model would be highly desirable. Our group recently reported on the development of a 3D cell culture system in which 3T3-L1 mouse preadipocytes or human orbital fibroblasts (HOF) were used as disease models for Graves’ orbitopathy (GO)^13^, and the deepening of the upper eyelid sulcus (DUES) induced by the use of prostaglandin analogues (PGs)^14,15^. In our earlier pilot study using this methodology, we successfully obtained 3D HTM spheroids, and found that the TGFβ2-induced the formation of significantly smaller and stiffer 3D HTM spheroids^16^. In addition, such TGFβ2-induced effects were substantially reduced by the presence of Rho-associated coiled-coil containing protein kinase (ROCK) inhibitors. Based on these findings, we concluded that 3D cultures using HTM and TGFβ2 may be a physiologically relevant model for POAG. Alternatively, since it was revealed that DEX treated 2D cultured HTM cells were also used for a model of SG^8^, we were prompted to determine whether our developed 3D culture method might also replicate an ex vivo SG model by using DEX.

In the current study, to establish a physiologically relevant in vitro models for POAG and SG by 3D cultures using HTM, we examined the effects of TGFβ2 or DEX on the size, morphology and physical properties of the 3D spheroids and the expression of ECM in these spheroids were compared with each other.

## Materials and methods

### Human trabecular meshwork (HTM) cells

All experiments involving human tissue/cells were performed in compliance with the tenets of the Declaration of Helsinki. Approval from the internal review board of Sapporo Medical University was obtained for the procurement and use of human eye tissue that was used in the study. Informed consent was obtained from all subjects or, if subjects are under 18, from a parent and/or legal guardian. Commercially available certified immortalized HTM cells that had been transfected with an original defective mutant of the SV40 virus (Applied Biological Materials Inc., Richmond Canada, product datasheets are attached in supplemental file) were used in the present study. To ensure that these HTM cells are truly TM cells, the DEX induced up-regulation in the mRNA expression of myocilin and extra domain A (EDA) fibronectin was confirmed among the criteria described in the consensus recommendations for TM cells as described by Keller et al.^17^ (Supplemental Fig. 1), although such DEX-induced up-regulation in the expression of myocilin and EDA fibronectin mRNA were not observed in human conjunctival fibroblasts (HconFs, ScienCell Reserch laboratories, CA USA) and human retinal pigment-epithelium (HRPE, ATCC, VA USA).

### 2D cultures of HTM cells

2D cultures of the HTM cells were prepared as described in a previous report^16^. Briefly, HTM cells, which were used after 20th passages, were maintained in 150 mm 2D culture dishes at 37 °C in 2D culture medium (HG-DMEM containing 10% FBS, 1% L-glutamine, 1% antibiotic–antimycotic) until reaching 90% confluence by changing the medium every other day. These HTM cells that were prepared as above were further processed for 3D spheroid preparation or transendothelial electron resistance (TEER) and fluorescein isothiocyanate (FITC) dextran permeability experiments described below.

### 3D culture of HTM cells

The 3D spheroids of HTM were generated by a hanging droplet spheroid three-dimension (3D) culture system as described in a previous report^13,14^. After collecting and resuspending the 2D cultured HTM cells as above, the 3D spheroid culture was processed on a hanging droplet spheroid (3D) culture plate (# HDP1385, Sigma-Aldrich) in the 3D spheroid medium (2D culture medium supplemented with 0.25% methylcellulose) during 6 days. In each well of the plate, 20,000 HTM cells were contained in 28 µl of the 3D spheroid medium. At Day 1, 250 nM DEX or 5 ng/mL TGFβ2 was supplemented, and half of the medium (14 μL) in each well was exchanged on every following day. The concentration of DEX was determined by the effectiveness of the response on their myocilin gene up-regulation, and that for TGFβ2 was determined as described in our previous study^16^.

### TEER measurements and FITC dextran permeability of 2D cultured HTM monolayer

The TEER and FITC dextran permeability measurements on HTM cell monolayers were carried out according to previously described methods^18,19^. Briefly, HTM cells prepared in 150 mm 2D cultured dishes as above were washed with phosphate buffered saline (PBS), and the cells were detached using 0.25% Trypsin/EDTA. After centrifugation for 5 min at 300×*g*, the cell pellet was re-suspended in 2D culture medium and HTM cells were seeded on 12 well plates for TEER (0.4 μm pore size and 12 mm diameter; Corning Transwell, Sigma-Aldrich) measurments at a density of 2.0 × 10^4^ cells per well. In each well of the TEER plate, the apical side (inside of the membrane inserts) and basal side (outside of the membrane inserts) were maintained in 0.5 mL and 1.5 mL of 2D culture medium, respectively. At Day 1, 250 nM DEX or 5 ng/mL TGFβ2 was added to the apical side of the 2D culture medium, and the culture medium of the apical side in each experimental group was changed every other day. TEER (Ωcm^2^) values were measured at day 6 using an electrical resistance system (KANTO CHEMICAL CO. INC., Tokyo, Japan) according to the manufacturer’s instructions after washing twice with PBS.

Concerning fluorescein isothiocyanate (FITC)-dextran permeability, 50 μmol/L of FITC-dextran (Sigma-Aldrich) was added to the well basal compartments of the culture and the culture medium from the apical compartment was collected at 60 min. The concentrations of the FITC-dextran were measured using a multimode plate reader (Enspire; Perkin Elmer, MA USA) at an excitation wavelength of 490 and an emission wavelength of 530 nm. The fluorescence intensity of the control medium was used as the background concentration.

### Scanning electron microscopy analysis of 2D or 3D HTM cells

2D HTM cells on the membrane for TEER or 3D HTM spheroids were prepared as above and fixed with 2.5% glutaraldehyde overnight, washed with PBS and processed for scanning electron microscopy (EM) using a HITACHI S-4300 microscope operated at 5 keV (the detector features 1280 × 960 pixel) according manufacturers’ operating protocol.

### Quantitative PCR

Total RNA extraction followed by reverse transcription and real-time PCR, and the quantification of respective genes normalized by comparing with the expression of housekeeping gene 36B4 (*Rplp0*) were described previously^16^. Sequene information of primers and Taqman probes used in the present study are shown in Supplemental Table 1.

### Immunocytochemistry of 2D cultures HTM cells and 3D HTM spheroids

Immunocytochemistry of the 2D cultured HTM cells and 3D HTM spheroids was examined by previously described methods, with minor modifications^14,15^. All procedures were performed at room temperature unless otherwise stated. Briefly, 2D cultured HTM cells on the slide glass (Lab-Tek II Chamber slide, Thermo Fisher Scientific Inc.) or 3D HTM spheroids prepared as above under several experimental conditions were fixed in 4% paraformaldehyde in PBS overnight, blocked in 3% BSA in PBS for 3 h, washed twice with PBS for 30 min. Then, those were reacted with an anti-human COL1, COL4, COL6 or FN rabbit antibody (1:200 dilutions) at 4 °C overnight. After washing 3 times with PBS for 1 h each, those were then reacted with 1:1000 dilutions of a goat anti-rabbit IgG (488 nm), phalloidin (594 nm) and DAPI for 3 h, and thereafter mounted with ProLong Gold Antifade Mountant with a cover glass. Immunofluorescent images were obtained by a Nikon A1 confocal microscopy using a × 20 air objective with a resolution of 1024 × 1024 pixels. For 3D spheroids, serials-axis images with a 2.2 μm interval during 35 μm height from their surface were obtained. The maximum intensity/surface area among above the observed areas was calculated using Image J (NIS-Elements 4.0 software) as follows: surface area = D × A/(A + π × H2), where D (μm) indicates spheroid diameter, A (μm^2^) indicates area of sectioned spheroid, and H (μm) indicates height (= 35 μm). For estimating the numbers of cells within a 3D spheroid, the volume of a 3D spheroid and the volume of a representative cell was calculated by assuming a spherical shape and the tentative diameters were estimated by largest cross-section of phalloidin images of the 3D spheroid (n = 5) and distance between two adjacent nuclei stained by DAPI (n = 5 for one section and was repeated five times using different preparations), respectively.

### Characterization of physical properties, sizes and stiffness, of the 3D HTM spheroid

The configuration of the 3D HTM spheroids was observed by phase contrast (PC, Nikon ECLIPSE TS2; Tokyo, Japan), and measurement of the mean size of each 3D organoid defined using the largest cross-sectional area (CSA) of the PC image was analyzed using the Image-J software version 1.51n (National Institutes of Health, Bethesda, MD) as described previously^13,14^.

The solidity of the 3D spheroids was measured using a micro-squeezer (MicroSquisher, CellScale, Waterloo, ON, Canada) equipped with a microscale compression system composed of a 406 μm diameter cantilever as recently reported^13^. A single spheroid placed on a 3-mm × 3-mm plate was compressed to a 50% deformation, as determined by a microscopic camera, for 20 s. The force required to achieve a 50% strain was measured through the cantilever, and the data are expressed as force/displacement (μN/μm).

### Statistical analysis

All statistical analyses were processed using the Graph Pad Prism 8 (GraphPad Software, San Diego, CA) were described previously^16^. Briefly, for the analysis of the difference between groups, a grouped analysis with a two-way analysis of variance (ANOVA) followed by Tukey’s multiple comparison test was used. Data are presented as arithmetic means ± standard error of the mean (SEM).

## Results

### Scanning electron microscopic, transendothelial electrical resistance (TEER) analyses and FITC dextran permeability measurement of the TGFβ2 or DEX treated 2D culture of HTM monolayer, and the mRNA expressions of ECM

**Figure 1:**
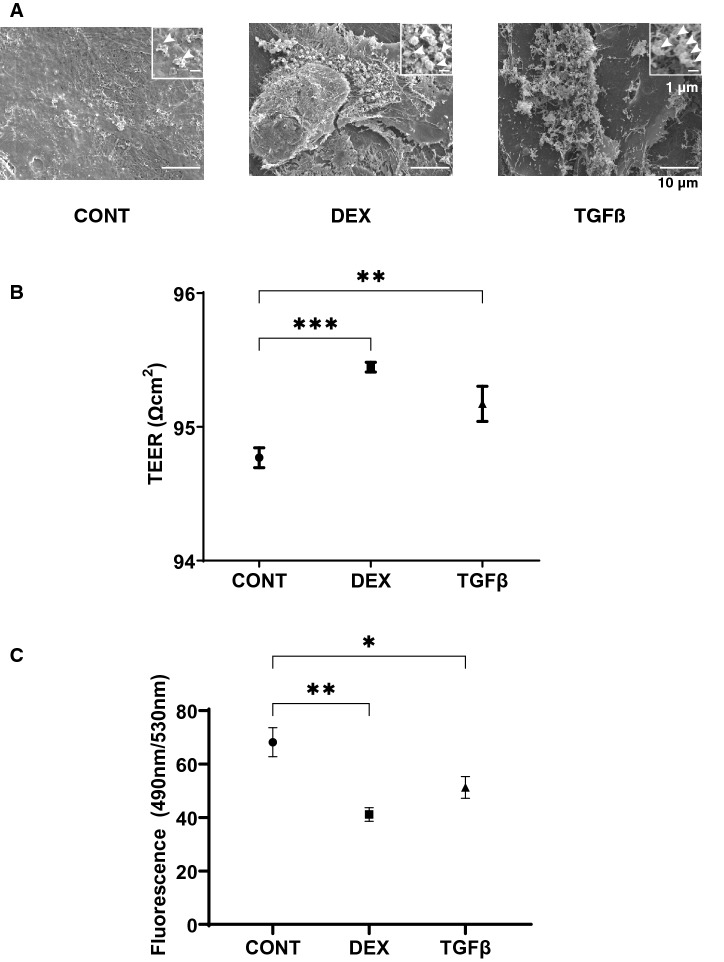
Representative scanning electron microscopic (SEM) images (**A**), transendothelial electrical resistance (TEER) (**B**) and FITC-dextran permeability (**C**) of 2D culture of HTM cell monolayer. Among the experimental groups treated without (CONT) or with 250 nM DEX or 5 ng/ml TGFβ2 (TGFβ), representative SEM images of 2D cultures HTM monolayer at Day 6 are shown (panel **A**, scale bar; 10 µm). Extracellular ECM deposits indicated by asterisks within inset of SEM images (scale bar; 1 µm) and numbers of ECM deposits within 5 × 5 µm^2^ (n = 5 different areas from 4 different preparations) were counted (CONT; 2.75 ± 1.47, DEX; 4.25 ± 0.83, TGFβ; 5.75 ± 0.83). To evaluate barrier function (Ω cm^2^) and the permeability of the 2D cultured HTM monolayers, TEER (panel **B**) and FITC-dextran permeability (panel **C**) measurements were made, respectively. All experiments were performed in triplicate using fresh preparations (n = 4). All data are presented as the arithmetic mean ± standard error of the mean (SEM). * *P* < 0.05, ** *P* < 0.01, *** *P* < 0.005 (ANOVA followed by a Tukey’s multiple comparison test).

**Figure 2:**
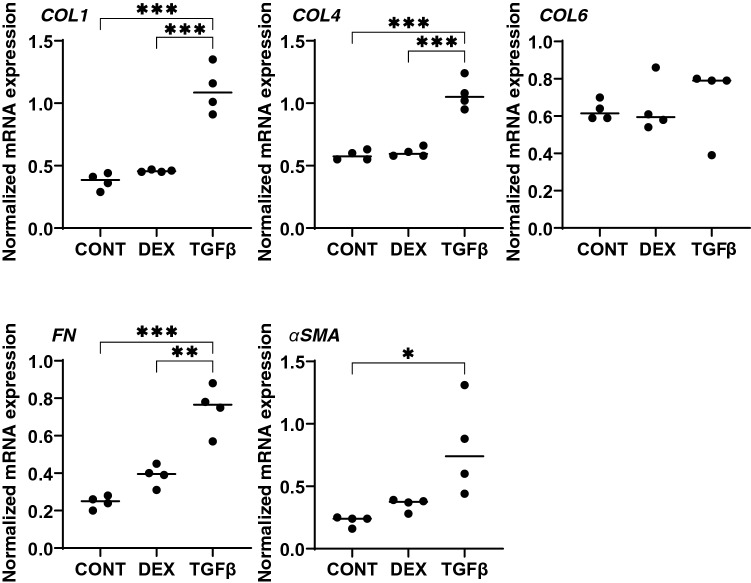
mRNA expression of ECMs in 2D cultured HTM cells. Among the experimental groups treated with or without 250 nM DEX or 5 ng/ml TGFβ2, 2D cultured HTM cells at Day 6 were subjected to a qPCR analysis to estimate the expression of mRNA of ECMs including *COL 1*, *COL 4*, *COL 6*, *FN* and *a-SMA*. All experiments were performed in duplicate using fresh preparations (n = 4). Data are presented as the arithmetic mean ± standard error of the mean (SEM). *P < 0.05; **P < 0.01; ***P < 0.005 (ANOVA followed by a Tukey’s multiple comparison test).

**Figure 3:**
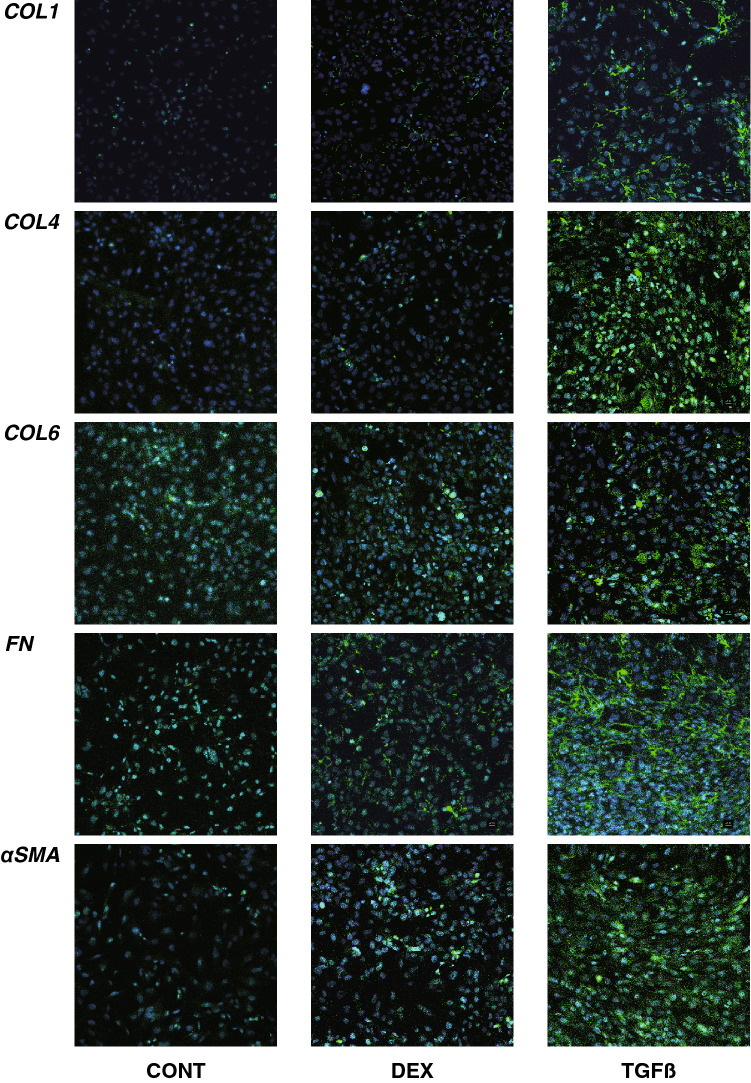
Immunolabeling of ECMs of the 2D cultured HTM cells. Among the experimental groups treated with or without 250 nM DEX or 5 ng/ml TGFβ2, 2D HTM cells at Day 6 were subjected to an immunostaining of *COL 1*, *COL 4*, *COL 6*, *FN* and *a-SMA*. All experiments were performed in duplicate using fresh preparations (n = 4). Demonstrative images are shown (ECM: green, DAPI: blue).

Effects of DEX and TGFβ2 on the morphology, barrier function and permeability of the 2D cultured HTM monolayers were studied by scanning electronic microscopy (SEM), transendothelial electron resistance (TEER) and FITC dextran permeability, respectively. As shown in Fig. 1A, the ultrastructure determined by SEM indicated that the ECM deposits (designated by arrows within insets) on the 2D cultures of HTM cell monolayer at Day 6 were significantly increased upon exposure to a 250 nM solution of DEX or to a 5 ng/ml solution of TGFβ2. Consistent with these morphological observations, the TEER values (panel B) and FITC dextran permeabilities (panel C) were substantially increased and decreased, respectively upon the administration of DEX or TGFβ2, and no significant difference was observed between DEX and TGFβ2. To elucidate the underlying mechanism causing these effects by DEX and TGFβ2, the mRNA expression of major ECMs comprised of HTM including COL1, 4 and 6, FN and α-SMA were evaluated. As shown in Fig. 2, the mRNA expressions of five major ECMs, except for COL6, were significantly up-regulated in the presence of TGFβ2, although DEX did not induce significant alterations. Such significant expressions of all ECMs except COL6 were also confirmed by immunohistochemistry (Fig. 3).

### Physical properties including size, morphology and stiffness of the 3D HTM spheroids treated by DEX or TGFβ2

**Figure 4:**
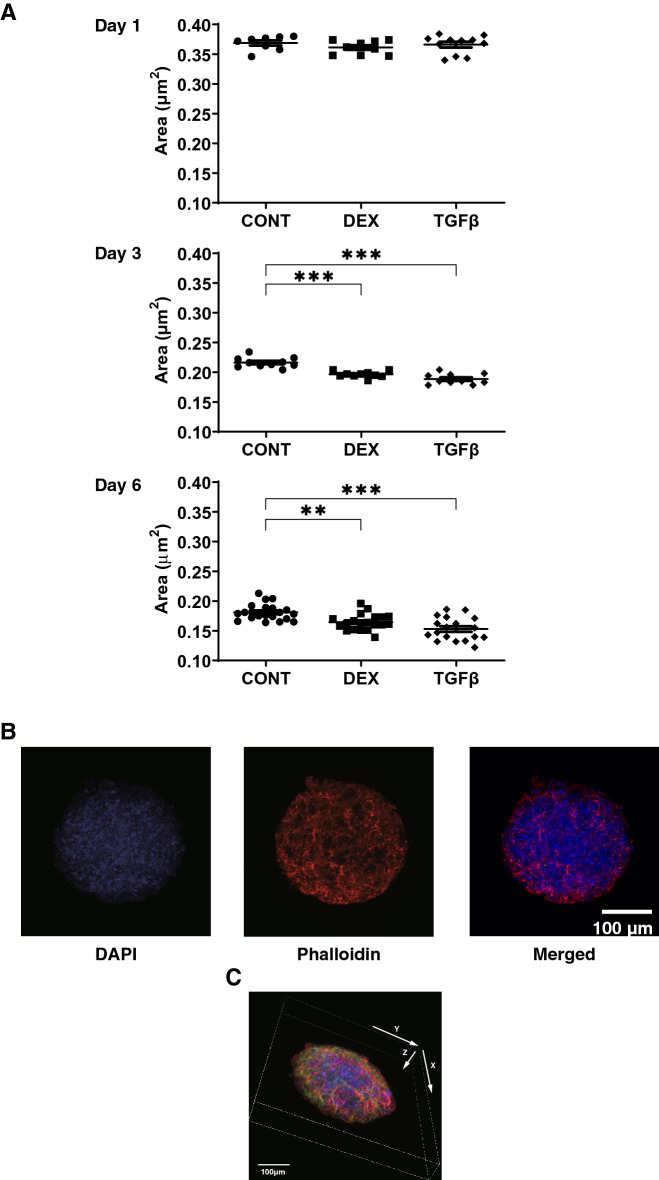
Changes in 3D HTM spheroid size (**A**) during their 3D culture in the presence or absence of DEX or TGFβ2, and representative 3D spheroid images stained by DAPI and phalloidin (**B**, **C**). Among the experimental groups treated without (CONT) or with 250 nM DEX or 5 ng/ml TGFβ2, changes in the mean size of 3D HTM spheroids during 3D culture at Days1, 3 and 6 were plotted in panel **A**. Representative immunolabeling XY axes plane image (panel **B**) and 3D XYZ axes image (panel **C**) of the 3D HTM spheroid (CONT) at Day 6 stained by DAPI and phalloidin. These experiments were performed in triplicate using fresh preparations (n = 10 or 5 for size measurement and immunolabeling, respectively). Data are presented as the arithmetic mean ± standard error of the mean (SEM). **P < 0.01; ***P < 0.005 (ANOVA followed by a Tukey’s multiple comparison test). Scale bar: 100 µm.

Since it is well known that human TM is composed of multiple layers of sheets^12^, a 3D drop culture method^20^ was employed to establish suitable models replicating glaucoma TM. In fact, as shown in Fig. 4A, as the maturation of the 3D HTM spheroids advanced during the 6-day culture, their sizes became smaller, consistent with the formation of normal 3D spheroids as observed in our previous studies using several types of adipocyte cells^13–15^ in addition to HTM^16^. Such down-sizing effects were further enhanced by DEX and TGFβ2, in which the effects of the latter were more evident. To confirm that these down-sizing processes were not artifacts or cell death within the inside of the 3D spheroid, they were stained with DAPI (Fig. 4B, C, and Supplemental Movie). Interestingly, as shown in Fig. 4B, multiple layers of HTM cells that were arranged concentrically were observed within the 3D HTM spheroid. Since intercellular interactions of the 3D spheroids were much stronger than that of 2D cultured cells^15^, it was impossible to count the numbers of cells present in the 3D spheroid. Thus, the numbers of cells in the 3D spheroid were estimated by calculating the volume of the overall 3D spheroid with the volumes of the cells within it, as calculated by cross-section images. The results indicated that approximately, the cell numbers of each 3D spheroid at Day 6 among three conditions (CONT; 21,545.1 ± 3748, DEX; 24,626.02 ± 5039, TGFβ; 29,539 ± 2362) were almost identical to those that were initially harvested (approximately 20,000 cells).

**Figure 5:**
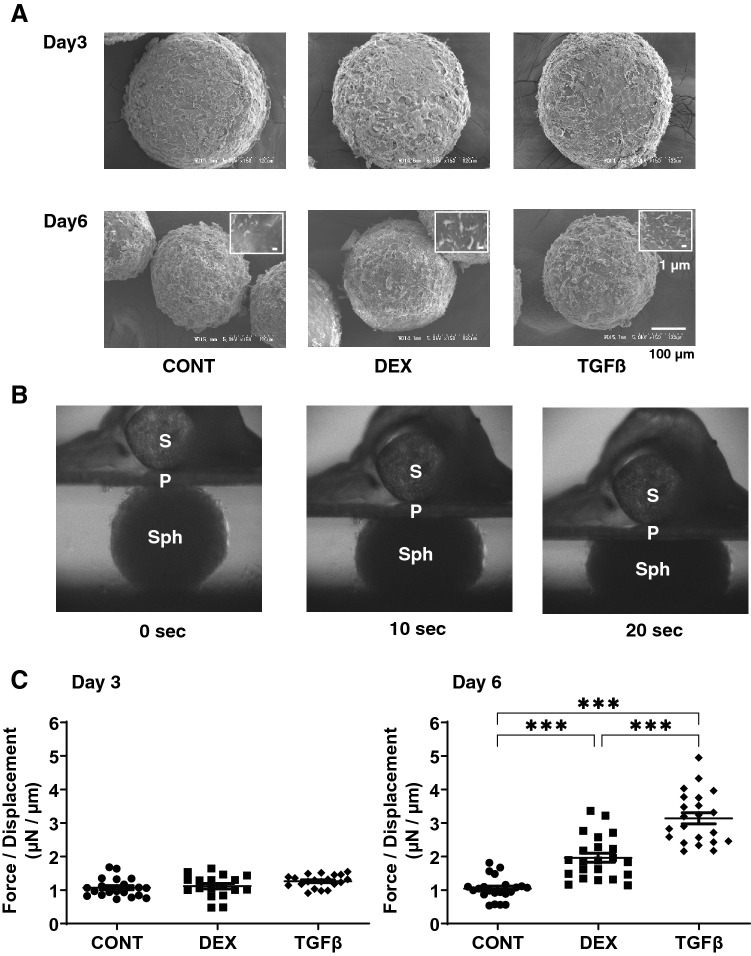
Ultrastructure and physical solidity of 3D HTM spheroids. Among the experimental groups treated with or without 250 nM DEX or 5 ng/ml TGFβ2, representative SEM images of 3D HTM spheroids at Day 3 or Day 6 are shown in Panel **A** (scale bar: 100 µm) and the physical solidity of their 3D spheroids at Day 3 and 6 was examined by a micro-squeezer. Numbers of ECM deposits of the 3D spheroid at Day 6 within 5 × 5 µm^2^ (panel **A** inset; scale bar: 1 µm) were counted in 5 different areas from 4 different preparations (CONT; 4.75 ± 0.83, DEX; 7.25 ± 0.83, TGFβ; 10.25 ± 2.05). Regarding the micro-squeezer analysis among experimental groups, the force (μN) required to induce a 50% deformity of every single out of 15–20 freshly prepared 3D spheroids were measured over a period of 20 s (Panel **B**; Sph: 3D spheroid, S: pressure sensor, P: compression plate) and force/displacement (μN/μm) values were plotted in Panel (**C**). Data are presented as the arithmetic mean ± standard error of the mean (SEM). ***P < 0.005 (ANOVA followed by a Tukey’s multiple comparison test).

To further study the effects of DEX and TGFβ2 on the physical properties of the 3D HTM spheroids, morphological evaluations by SEM and physical stiffness analysis by a micro-squeeze device were performed (Fig. 5). SEM indicated that there was a significant increase in of ECM deposits on the surface (panel A) and that the stiffness of the 3D HTM spheroid (panels B and C) was increased in the presence of DEX or TGFβ2, and these effects were more evident in the case of TGFβ2. Since this stiffness analysis using a micro-squeezer is the only reasonable method for evaluating the physical stiffness of the single living 3D spheroid^13^ and our 3D HTM spheroids consisted of multiple layers of concentric lined HTM cells as above, we assumed that these results reflects the hardness of whole structures of in vivo multiple layers of human TM.

### mRNA expressions of ECM of the 3D HTM spheroid treated by DEX or TGFβ2, and effects of TIMPs and MMPs toward 2D and 3D cultured HTM cells

**Figure 6:**
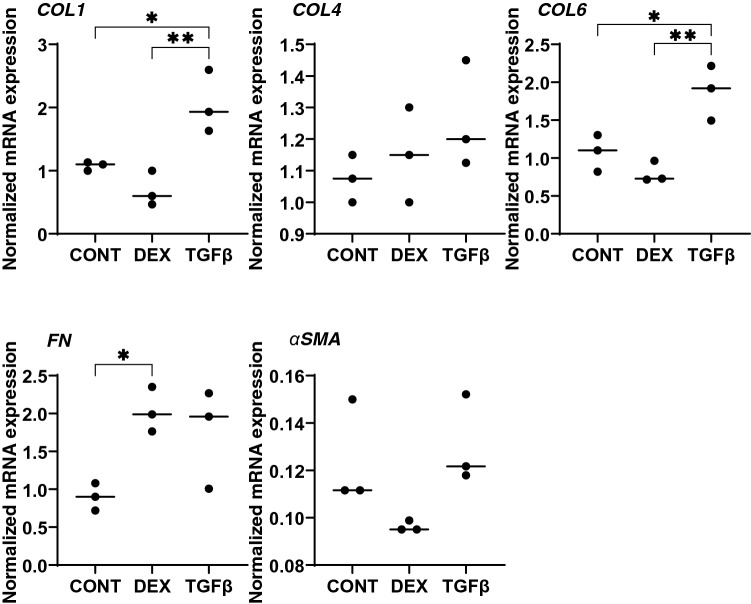
mRNA expression of ECMs in 3D cultured HTM spheroids. Among the experimental groups treated with or without 250 nM DEX or 5 ng/ml TGFβ2, 3D HTM spheroids at Day 6 were subjected to qPCR analysis to estimate the expression of mRNA in ECMs including *COL 1*, *COL 4*, *COL 6*, *FN* and *a-SMA*. All experiments were performed in duplicate using fresh preparations (n = 3). Data are presented as the arithmetic mean ± standard error of the mean (SEM). *P < 0.05; **P < 0.01 (ANOVA followed by a Tukey’s multiple comparison test).

**Figure 7:**
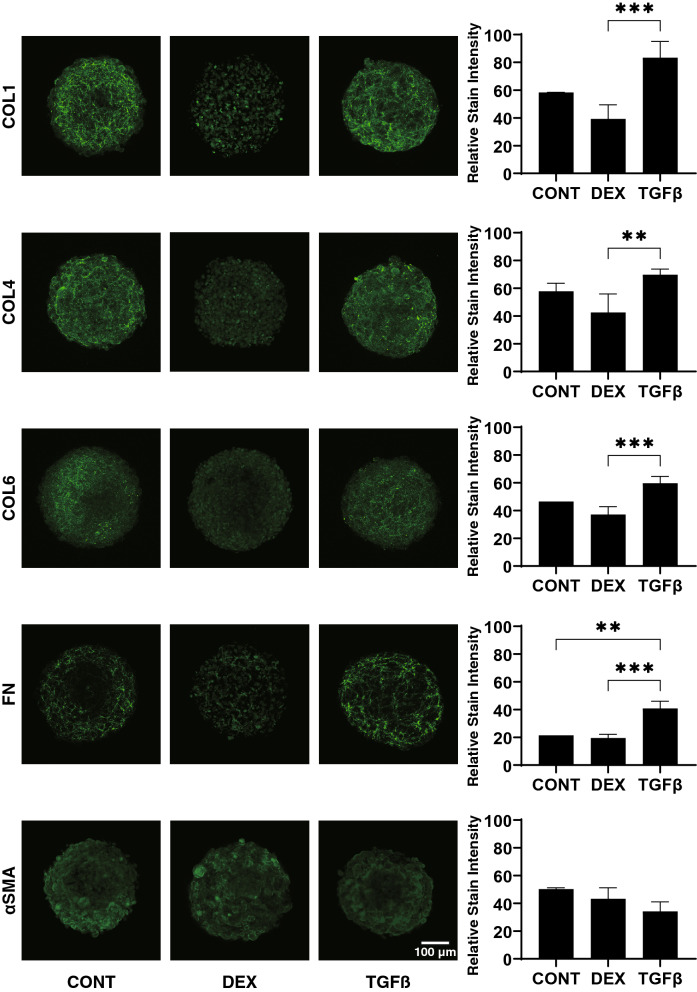
Immunolabeling of ECMs of the 3D HTM spheroids. Among the experimental groups treated with or without 250 nM DEX or 5 ng/ml TGFβ2, 3D HTM spheroids at Day 6 were subjected to immunostaining for *COL 1*, *COL 4*, *COL 6*, *FN* and *a-SMA*. All experiments were performed in duplicate using fresh preparations (n = 4). Representative XY axes plane images are shown in left panels and relative staining intensities were plotted in right panels. Data are presented as the arithmetic mean ± standard error of the mean (SEM). **P < 0.01; ***P < 0.005 (ANOVA followed by a Tukey’s multiple comparison test). Scale bar: 100 µm.

Regarding the mRNA expression of ECMs (Fig. 6), upon administering a 5 ng/ml solution of TGFβ2, COL1 and 6 were significantly up-regulated. In contrast, 250 nM DEX induced a significant up-regulation in the expression of FN. However, in contrast, the immunostaining of the 3D spheroids indicated that only FN expression was significantly increased by TGFβ2 (Fig. 7). Such a discrepancy between mRNA expression and immunostaining levels of the 3D HTM spheroids was also observed in our previous study^16^. As possible reason for the discrepancy, the possible involvement of several post-translational modifications and proteolysis by several proteolytic enzymes including MMPs, in addition to spatial localization within the 3D spheroid^16^ cannot be excluded.

**Figure 8:**
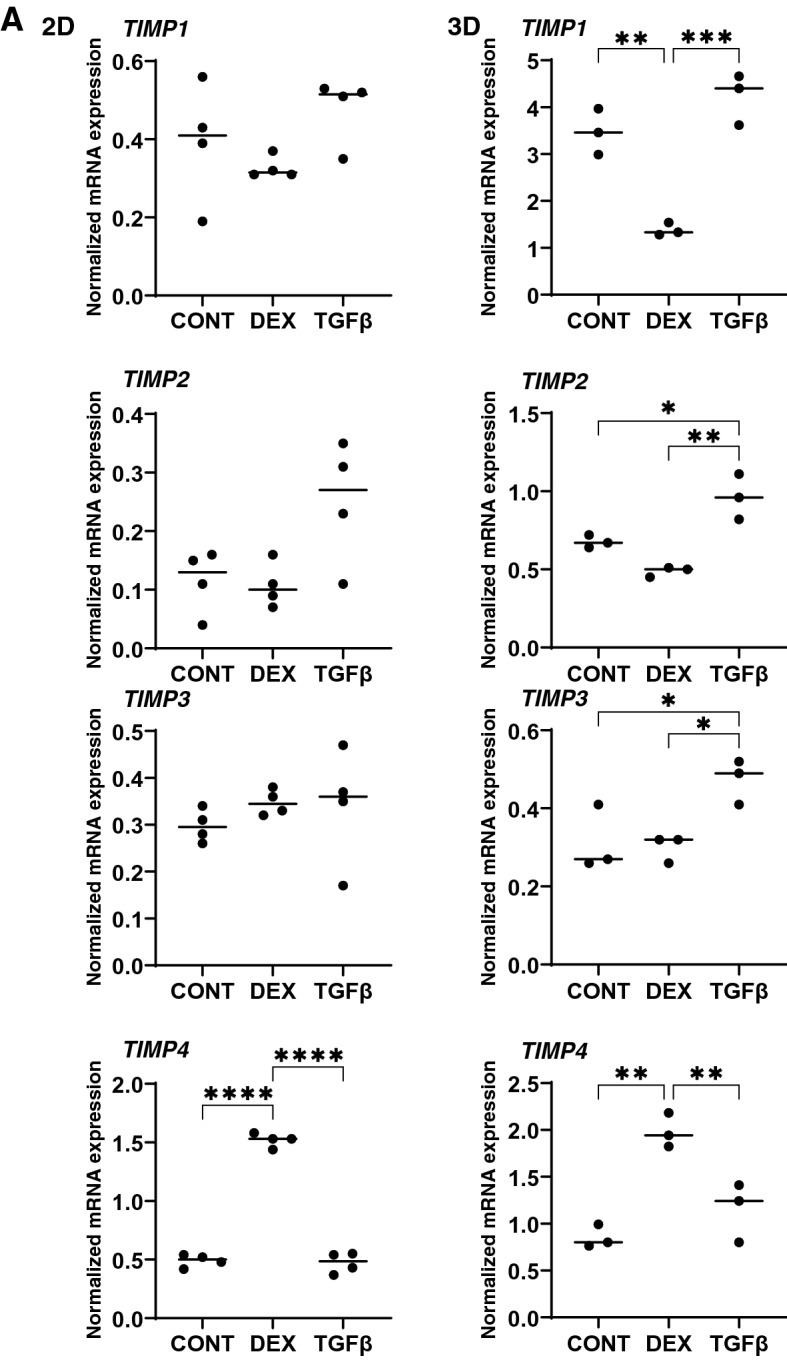

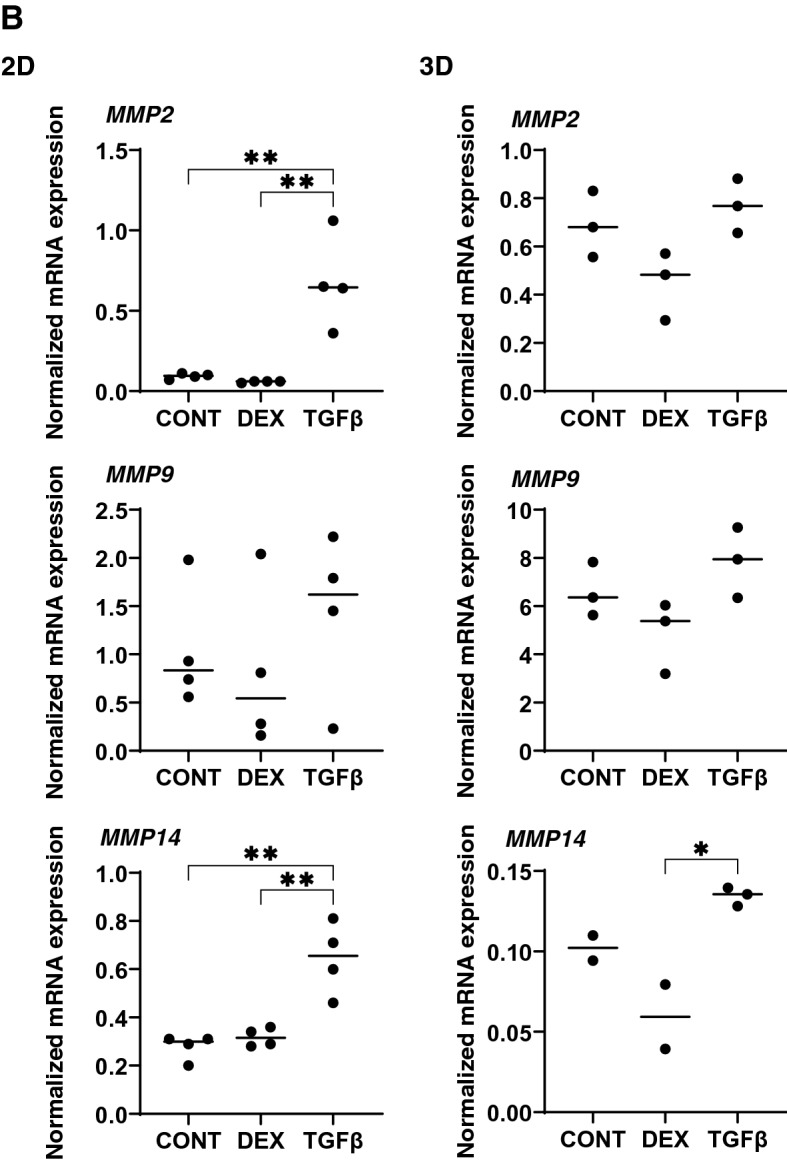
mRNA expression of TIMPS and MMPs in HTM 2D cells and 3D spheroids. Among the experimental groups treated with or without 250 nM DEX or 5 ng/ml TGFβ2, HTM 2D cells and 3D spheroids at Day 6 were subjected to qPCR analysis to estimate the expression of mRNA in *TIMP 1–4* (panel **A**), and *MMP2,9 and 14* (panel **B**). All experiments were performed in duplicate using fresh preparations (n = 3). Data are presented as the arithmetic mean ± standard error of the mean (SEM). *P < 0.05; **P < 0.01; ***P < 0.005 (ANOVA followed by a Tukey’s multiple comparison test).

**Figure 9:**
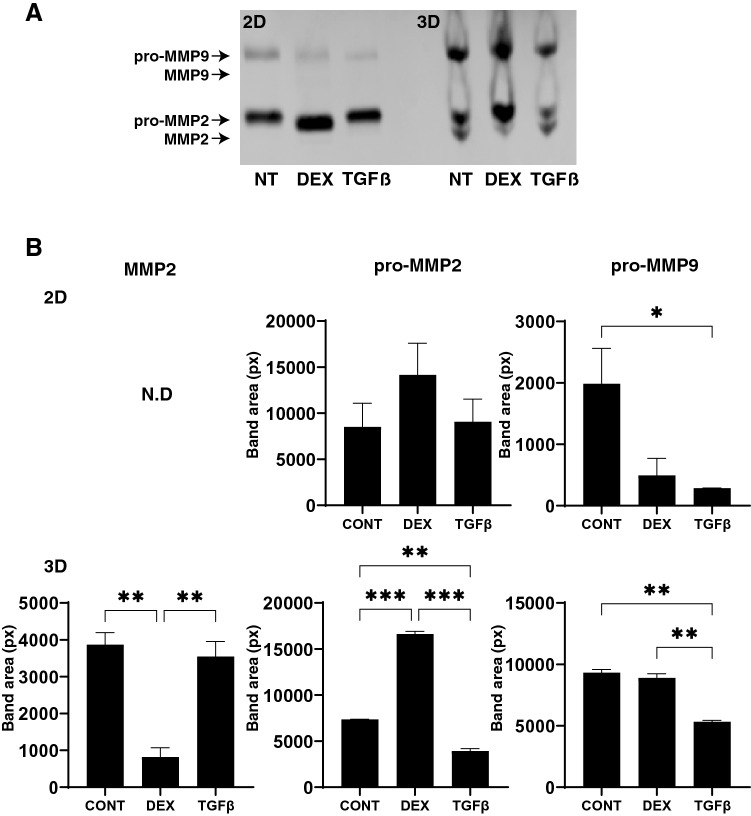
Zymography of HTM 2D cells and 3D spheroids. Among the experimental groups treated with or without 250 nM DEX or 5 ng/ml TGFβ2, HTM 2D cells and 3D spheroids at Day 6 were subjected to zymography measurements (panel **A**). The corresponding MMP band areas were evaluated using Image J soft (panel **B**). All experiments were performed in duplicate using fresh preparations (n = 3). Data are presented as the arithmetic mean ± standard error of the mean (SEM). *P < 0.05; **P < 0.01; ***P < 0.005 (ANOVA followed by a Tukey’s multiple comparison test).

To study this issue further, the mRNA expression of TIMPS and MMPs, well-known modulators of ECM metabolism, were evaluated. Upon the administration of DEX or TGFβ2, (1), mRNA expression (Fig. 8), we found a significant down-regulation of TIMP1 (3D) and an up-regulation of TIMP4 (2D and 3D), or a significant up-regulation of TIMP2 and 3 (3D) and MMP2 and 14 (2D), respectively and (2) zymography (Fig. 9); a down-regulation of MMP2 and an up-regulation of pro-MMP2 (3D), or a down-regulation of pro-MMP9 (2D and 3D), respectively.

## Discussion

The use of 3D spheroid cell cultures has recently received great attention for being suitable disease in vivo models for a variety of diseases^20^. In comparison with the intercellular side by side interactions in conventional 2D cell cultures, since each cell can interact with other cells in a 3D spatial space, the protein network of the surrounding the ECMs may be similar to those of in vivo organs and it would therefore appear that such 3D spheroids could replicate real tissues and the biological features associated with them^21^. For example, we previously focused on diseases that affect orbital fatty tissues in which adipocytes were grown within a 3D conic space, such as Graves’ orbitopathy (GO). Using the 3D drop-culture methods, we were able to successfully produce 3D spheroids from HOFs in patients with GO, and found that HIF2A played a pivotal role in the LOX-dependent ECM accumulation^13^. Furthermore, to elucidate the molecular pathology of the prostaglandin (PGs)-induced deepening of the upper eyelid sulcus (DUES), we also employed this 3D culture system using HOFs and 3T3L1 cells, and found that PGs significantly suppressed the sizes of the 3D spheroids, and modulated the spatial distribution of several ECMs that surround the 3D spheroids^14,15^. Based upon these collective findings, we propose that our developed 3D cell culture would be viable in vitro models of GO and DUES and would also be applicable for use in numerous other cells and organs.

Using a steroid-induced glaucoma model using a scaffold-assisted 3D HTM culture, Torrejon et al. previously reported that the overexpression of ECM, impaired HTM cell phagocytic activity and caused an increased resistance to outflow^22^. In addition, Vernazza et al. compared cellular responses after chronic stress exposure between the 2D and 3D cultured HTM, and found that 3D-cultured cells are more sensitive against intracellular reactive oxidative species production induced by a hydrogen peroxide treatment in comparison with conventional 2D cultures^23^. However, these 3D culture techniques may have some drawbacks with regard to mimicking the physiological and pathological conditions of human TM because of the presence of scaffolds which are missing in human TM. In contrast, however, since we recently successfully obtained 3D spheroids using human orbital fibroblasts (HOFs) by a 3D cell drop culture method in the absence of a scaffold^13–15^, we applied our unique 3D drop culture method to establish an in vitro model for glaucomatous HTM. In our earlier pilot study, we successfully obtained 3D HTM spheroids, and found that TGFβ2 significantly induced the down-sizing and stiffness of 3D HOF spheroids, and those effects were substantially inhibited by ROCK-i^16^.

ECM is an important multifunctional molecular group that is involved in structurally supporting organs as well as modulating cell–cell signals and regulating a variety of cellular functions^24^. Among the ECM, the most abundant are COL1, and COL4 and COL6 are major components of the basement membrane (BM)^25–28^. FN, composed of highly interwoven fibers, is involved in defining cell shape and contractility in association with COL1^29^. Within TM cells, TGFβ2 activates cytoplasmic Smad2/3^30,31^, which, in turn, leads to increased ECM expression, such as FN and COL4 as fibrotic changes. These changes can be involved in the TGFβ2–induced impediment to AH outflow through the TM, thus resulting in elevated IOP levels^32^. Similarly, the steroid-induced elevation of IOP is also generally thought to be caused by increased resistance to aqueous outflow through associated changes in the TM and its ECM^33–35^. In fact, the findings of several experimental studies, including scaffold assisted 3D cultures, indicated that the secretion of FN from TM cells is substantially enhanced upon treatment with corticosteroids^36–38^. In our present study, such corticosteroid induced up-regulation of FN was also observed in 3D HTM spheroids but not in 2D cultured HTM upon DEX exposure.

It was reported that ECM remodeling by MMPs enhances AH outflow through the TM in a study using perfused human anterior segment organ cultures^39^. Furthermore, a previous study demonstrated that the exposure of TM organ cultures to corticosteroids caused a decrease in the levels of MMP 9^40^. In addition, another study using human corneoscleral explant cultures containing both ciliary bodies and TM also indicated that exposure to dexamethasone led to a decreased activity of MMP2, MMP3, and MMP9 on zymography^41^. More recently, De Groef et al. reported that the remodeling of the TM by MMP9 is required to enhance the outflow and maintain IOP homeostasis based upon an altered structural organization of the TM and the occurrence of early-onset ocular hypertension in MMP9 knockout mice^42^. In our current study, the mRNA expression of TIMPs and MMPs by the DEX treated 3D HTM spheroids were quite different from those of TGFβ2 treated 3D HTM spheroids, suggesting that the mechanisms responsible for ECM remodeling by TIMPs and MMPs may be different between the TGFβ2 and DEX treatment conditions, although their physical properties, i.e., smaller and stiffer 3D spheroids, were similar. Alternatively, in a study in which the AH concentrations of MMPs and TIMPs were measured, the levels of MMP2 were significantly decreased in patients with POAG as compared to cataractous patients, while the levels of TIMP2 were unchanged^43^. In contrast, another similar study reported that the AH concentrations of MMP2 and TIMP2 levels were significantly increased in patients with POAG as compared with cataractous patients^44^. Thus, these collective results suggest that an imbalance between the MMP/TIMP ratio may contribute to the pathogenesis associated with the decreased outflow facility and elevated IOP in POAG^43,44^. In our current TGFβ2 treated POAG model of the 3D HTM spheroid, a significant up-regulation of TIMP2 and 3, while substantial up-regulation of TIMP4 was observed in DEX treated SG model. Therefore, these collective findings suggest that both of TGFβ2-treated or DEX-treated 3D HTM spheroids may rationally be applicable as in vitro POAG or SG models, respectively, for studies of the pathogenic conditions of the HTM observed in POAG or SG as well as the pharmacological effects of several anti-glaucoma drugs toward these HTM.

However, the present study has several limitations that need to be discussed; First, the current study was performed using commercially available immortalized HTM cells instead of primary HTM cells. Although the provider certified that these are truly HTM cells, it is known that there is significant biological variability from donor to donor, and therefore studying one HTM cell line would be insufficient to determine whether the effects seen are representative across donor tissues/cells. Unfortunately, under our national laws, we are not permitted to use human donor eyes for research. Given this situation, we tested for the DEX-induced up-regulation in myocilin or EDA-FN expression of these cells before use (Supplemental Fig. 1), and such effects were not observed in either HconF cells or in HRPE cells. Furthermore, our current study approach to replicate in vitro different models for glaucomatous HTM, that is, POAG and SG, using a 3D culture technique should still have some value in terms of glaucoma-related research. Therefore, an additional study using confirmed glaucomatous as well as non-glaucomatous HTM cells without any glaucomatous stimulus like TGFβ2 and/or DEX from several different human donors will be needed. Second, our present data indicated that the physical properties of the 3D HTM spheroids were significantly modulated by the presence of TGFβ2 or DEX, and suggested that those may replicate multiple sheet structures of POAG or SG related TMs. Third, several different observations such as mRNA expression of gene expressions were observed between 2 and 3D cultures. Although the possible mechanisms causing these differences have not been elucidated at present, several different biological properties between 2 and 3D cultures were also recognized regarding the adipogenesis of preadipocytes of the 3T3-L1 cells^15^, in addition to the ECM expression by immunohistochemistry and gene expressions of HTM cells^16^. However, our 3D spheroid models may not mimic the key functions of glaucomatous TM cells, such as resistance to outflow at present. To determine this outflow resistance property of our 3D spheroid models, additional analyses will also be required. Fourth, in the SEM images of the 2D and 3D HTM cell cultures, we assumed that the deposits marked by arrow heads in Figs. 1A and 5A based upon previous observations of SEM images were, in fact, ECM deposits^45^. However, at present, we have not yet confirmed that these ECM deposits were derived from ECM components. Therefore, to further characterize this issue, additional analyses using immunoelectron microscopy will be required. Lastly, regarding the structural similarity between our 3D HTM spheroids and in vivo TM structures, we assume that in our 3D HTM spheroids, the HTM cells are lined up concentrically and that multiple layers are then formed, although this may be somewhat different from the “multiple sheet layers” found in human TM structures. Interestingly, in our previous study using 3D preadipocyte spheroids, cell to cell interactions were much more evident as compared to those in 2D culture based upon trypsin sensitivity analysis^15^. Thus, we suggest that this may be ascribed to inter-concentrical interactions between layers, in addition to intercellular interactions within a layer. However, this is also speculative at present, if our speculations are correct, some cell adhesion factors may be related. Therefore, further investigations into this subject using our new 3D culture models of POAG or SG using confirmed glaucomatous TM cells will clearly be needed and ex vivo the use of anterior segment and mouse models will be our next focus.

## Supplementary Information

Supplementary Information 1.

Supplementary Video 1.

Supplementary Legends.
