# Supplementary material for: Establishment of appropriate glaucoma models using dexamethasone or TGFβ2 treated three-dimension (3D) cultured human trabecular meshwork (HTM) cells
Source: Sci Rep. 2021 Sep 29;11:19369. doi: 10.1038/s41598-021-98766-3 (PMC8481525; doi:10.1038/s41598-021-98766-3)
Supplement: Supplementary file 1 — Supplementary Information 1. [file 41598_2021_98766_MOESM1_ESM.pdf]

A

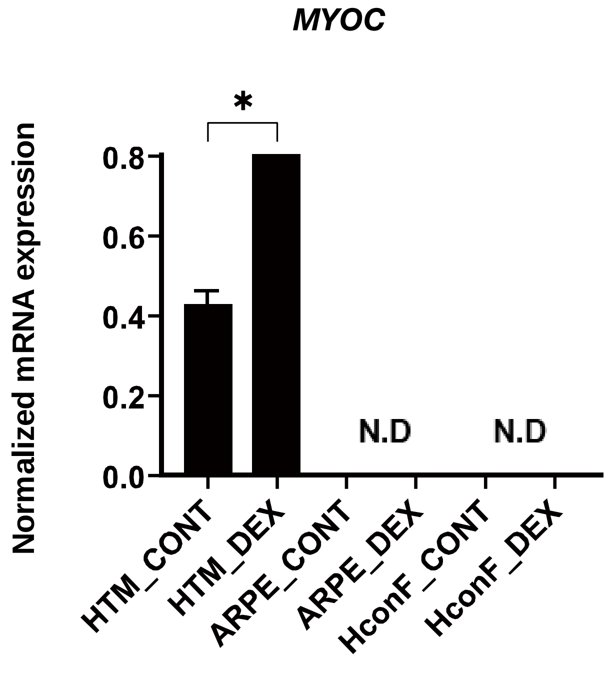

B

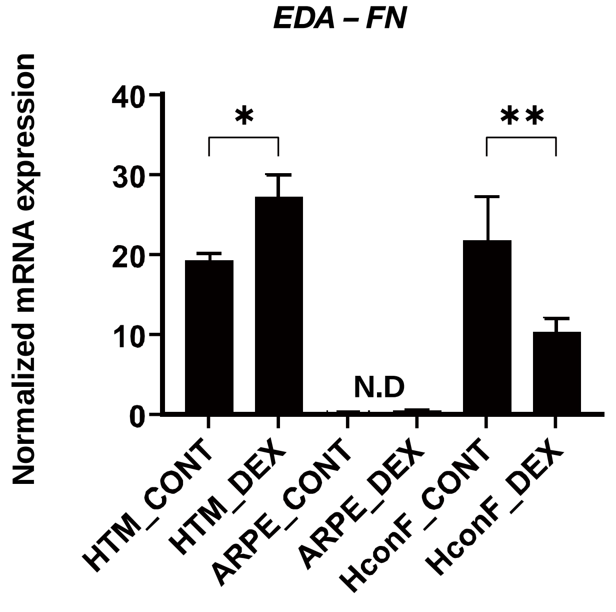

Supplemental Figure 1 mRNA expression of myocilin or EDA fibronectin in 2D cultured HTM, human conjunctival fibroblast (HconF) or human retinal pigment epithelium (HRPE).

In the absence or presence of 250 nM DEX, 2D cultured HTM, HconF or

HRPE at Day 6 were subjected to qPCR analysis to estimate the expression of mRNA of myocilin (*MYOC*, panel A) or EDA fibronectin (*EDA-FN*, panel B). All experiments were performed in duplicate using fresh preparations. Data are presented as the arithmetic mean ± standard error of the mean (SEM). * P<0.05, ** P<0.01 (ANOVA followed by a Tukey's multiple comparison test).

		Sequence	Exon Location	RefSeqNumber
human RPLP0	Probe	5'-/56-FAM/CCCTGTCTT/ZEN/CCCTGGGCATCAC/3IABkFQ/-3'	2-3	NM_001002
	Primer2	5'-TCGTCTTTAAACCCTGCGTG-3'		
	Primer1	5'-TGTCTGCTCCCACAATGAAAC-3'		
human COL1A1	Probe	5'-/56-FAM/TCGAGGGCC/ZEN/AAGACGAAGACATC/3IABkFQ/-3'	1-2	NM_000088
	Primer2	5'-GACATGTTCAGCTTTGTGGAC-3'		
	Primer1	5'-TTCTGTACGCAGGTGATTGG-3'		
human COL4A1	Probe	5'-/56-FAM/TCATACAGA/ZEN/CTTGGCAGCGGCT/3IABkFQ/-3'	51-52	NM_001845
	Primer2	5'-AGAGAGGAGCGAGATGTTCA-3'		
	Primer1	5'-TGAGTCAGGCTTCATTATGTTCT-3'		
human COL6A1	Primer2	5'-CCTCGTGGACAAAGTCAAGT-3'	2-3	NM_001848
	Primer1	5'-GTGAGGCCTTGGATGATCTC-3'		
human FN1	Primer2	5'-CGTCCTAAAGACTCCATGATCTG-3'	3-4	NM_212482
	Primer1	5'-ACCAATCTTGTAGGACTGACC-3'		
human αSMA	Probe	5'-/56-FAM/AGACCCTGT/ZEN/TCCAGCCATCCTTC/3IABkFQ/-3'	8-9	NM_001613
	Primer2	5'-AGAGTTACGAGTTGCCTGATG-3'		
	Primer1	5'-CTGTTGTAGGTGGTTTCATGGA-3'		
human TIMP1	Probe	5'-/56-FAM/TCAACCAGA/ZEN/CCACCTTATACCAGCG/3IABkFQ/-3'	2-4	NM_003254
	Primer2	5'-CCTTCTGCAATTCCGACCT-3'		
	Primer1	5'-GCTTGGAACCCTTTATACATCTTG-3'		
human TIMP2	Probe	5'-/56-FAM/TCTCATTGC/ZEN/AGGAAAGGCCGAGG/3IABkFQ/-3'	3-4	NM_003255
	Primer2	5'-GACGTTGGAGGAAAGAAGGA-3'		
	Primer1	5'-TGTGGTTCAGGCTCTTCTTC-3'		
human TIMP3	Probe	5'-/56-FAM/CCTCCTTTA/ZEN/CCAGCTTCTTCCCCAC/3IABkFQ/-3'	1-3	NM_000362
	Primer2	5'-CCTTCTGCAACTCCGACATC-3'		
	Primer1	5'-CGGTACATCTTCATCTGCTTGA-3'		
human TIMP4	Probe	5'-/56-FAM/ACTGAGGAC/ZEN/CTGACCAGTCAAGAGA/3IABkFQ/-3'	3-4	NM_003256
	Primer2	5'-GGTTTGAGAAAGTCAAGGATGTTC-3'		
	Primer1	5'-GTTGCACAGATGGATGAAGAC-3'		
human MMP2	Primer2	5'-TCCACCACCTACAACTTTGAG-3'	6-7	NM_004530
	Primer1	5'-GTGCAGCTGTCATAGGATGT-3'		
human MMP9	Primer2	5'-ACATCGTCATCCAGTTTGGTG-3'	3-4	NM_004994
	Primer1	5'-CGTCGAAATGGGCGTCT-3'		
human MMP14	Primer2	5'-TTCGCCGACTAAGCAGAAG-3'	1-1	NM_004995
	Primer1	5'-CTTGAATTCCTAGACCGCTGT-3'		
human MYOC	Probe	5'-/56-FAM/CCTCTCCAC/ZEN/TCCTGAGATAGCCAGA/3IABkFQ/-3'		
	Primer2	5'-CTGCTTCCCGAATTTTGAAGG-3'		
	Primer1	5'-ATCCACACACCATACTTGCC-3'		

Supplemental Table 1 Sequences of primers of qPCR

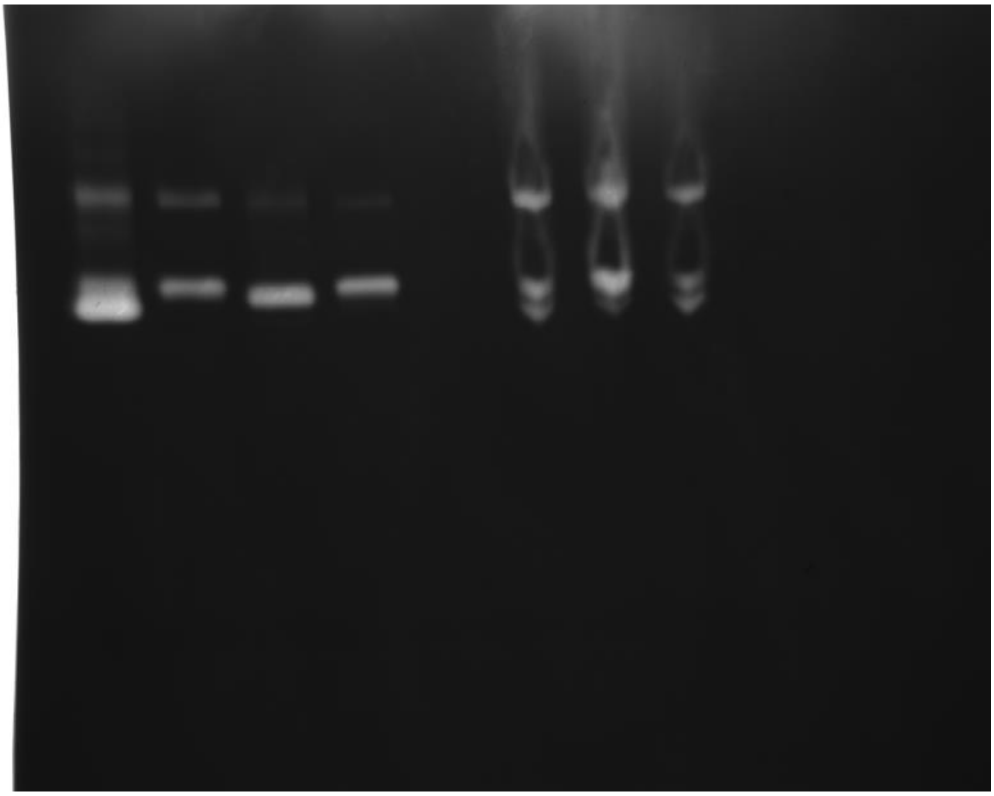

Supplemental information for zymography

Fig. 9 is the monochrome inversion image obtained from original zymography gel attached here.

Product Datasheet

abm

Immortalized Human Primary Trabecular Meshwork Cells - SV40

Cat. No.
T0371

Quantity
$1x10^6$ cells / 1.0 ml

**Organism**	Homo sapiens
**Source Organ**	Juxtacanalicular and corneoscleral regions of human eye
**BioSafety Level**	II
**Growth Properties**	Adherent
**Morphology**	Polygonal
**Recommended Seeding Density**	10,000 - 20,000 cells/cm^2
**Population Doubling**	18-25 hours
**Immortalization Method**	Serial passaging and transduction with recombinant lentiviruses carrying SV40 Large T antigen
**Description**	Immortalized Human Primary Trabecular Meshwork Cells - SV40 were derived from the normal juxtacanalicular and corneoscleral tissue regions of the human eye, and were immortalized with SV40. Recommended for use in research for glaucoma and studies involving trabecular meshwork functions.
**Applications**	For Research Use Only
**Propagation Requirements**	**Use of PriCoat™ T25 Flasks (G299) or Applied Cell Extracellular Matrix (G422) is required for cell adhesion to the culture vessels. Grow cells in ECM-coated culture vessels with the following conditions.** he base medium for this cell line is Prigrow III medium available at **abm**, Cat. No. TM003. To make the complete growth medium, add the following components to the base medium: fetal bovine serum (TM999)* to a final concentration of 10% and Penicillin/Streptomycin Solution (G255) to a final concentration of 1%. Carbon dioxide (CO_2): 5%, Temperature: 37.0°C. *** abm does not recommend to use heat-inactivated FBS for cell culture unless specified otherwise.**
**Procedure Overview**	

コスモ・バイオ株式会社 COSMO BIO CO., LTD. 研究用 X69

品 名 / Desc.	Immortalized Trabecular Meshwork Cells -	
メーカー / -	APB Applied Biological Materials Inc.	
品 番 /	T0371-C-ACADEMIC	
サ イ ズ / Size	1 EACH	1x10^6 cells / 1.0
ロ ッ ト / Lot	0057835530001	
法令区分 / -	カルタヘナ	
貯蔵温度 / Store	液窒[LN2]	
輸送条件 / Trans	ドライアイス多	

2000001838457

1-866-757-2414 (Toll Free)
604-247-2416 (Local)

Order: order@abmGood.com
Website: www.abmGood.com

Applied Biological Materials Inc.
8 - 13520 Crestwood Place,
Richmond, BC, CANADA V6V 2G2

Supplemental information for datasheet1 of immortalized HTM cell from company.

Certificate of Analysis

SUBMITTED BY:
Quality Control Personnel: 0017
Quality Assurance Personnel: 0130

Applied Biological Materials Inc.
1-3671 Viking Way,
Richmond, BC, CANADA
V6V 2J5

Product Description

**Product Name:**	Immortalized Human Primary Trabecular Meshwork Cells – SV40
**Cat. Number:**	T0371–C
**Lot Number:**	0057835530001
**Date of Manufacture:**	10/ 2018
**Species:**	*Homo sapiens*
**Source Organ:**	Eye
**Quantity:**	1 vial (Cryopreserved)
**Passage Number:**	12
**Cell Count:**	1.11 x10 ^{6} Cells/ml
**Viability:**	> 80%
**Population Doubling Time:**	18-25 hours
**Recommended Seeding Density:**	10,000 – 20,000 cells/cm^2
**Storage Conditions:**	-180°C

Specifications

Test	Method	Results
Sterility	****	Pass
Mycoplasma	PCR	Not Detected

Approved By:

Vidhu Sharma

Approved Date: October 12, 2018

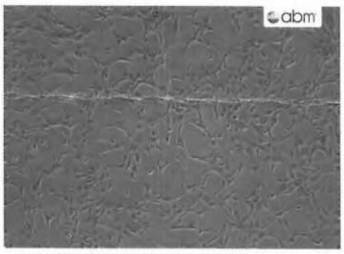

T0371 – Immortalized Human Primary Trabecular Meshwork Cells – SV40

This product is for research use only and is not intended for therapeutic or diagnostic applications.
Please contact a technical service representative for more information.

Supplemental information for datasheet2 of immortalized HTM cell from company.
