# Supplementary material for: Establishment of appropriate glaucoma models using dexamethasone or TGFβ2 treated three-dimension (3D) cultured human trabecular meshwork (HTM) cells
Source: Sci Rep. 2021 Sep 29;11:19369. doi: 10.1038/s41598-021-98766-3 (PMC8481525; doi:10.1038/s41598-021-98766-3)
Supplement: Supplementary file 3 — Supplementary Legends. [file 41598_2021_98766_MOESM3_ESM.docx]

**Supplementary Video 1.** Series of the confocal XY plane images along the Z axis of the 3D HTM spheroid (CONT) at Day 6 stained with DAPI (blue), COL 1 (green) and phalloidin (red).
